# A First Investigation on *Verbascum propontideum* Murb.: Comparative Biological Properties, Phytochemical Profile and Mineral Composition

**DOI:** 10.1007/s11130-026-01470-8

**Published:** 2026-02-17

**Authors:** Burcu Şahin, Mehlika Alper

**Affiliations:** Department of Molecular Biology and Genetics, Faculty of Sciences, Muğla Sıtkı Koçman University, Muğla, 48000 Kötekli Turkey

**Keywords:** *Verbascum propontideum*, Cell viability, Apoptosis, Antioxidant, Phenolic compound, Mineral composition

## Abstract

The current study revealed for the first time various health-related biological properties of different extracts of *Verbascum propontideum* Murb. Additionally, mineral element analyses were performed. Cell viability was evaluated by the MTT assay. Apoptotic cell death using the Annexin V/PI method and cell cycle analyses were conducted by flow cytometry. To investigate the antioxidant activities, different assays were performed. Quantitative analyses were performed. The phenolic compounds were quantified by HPLC analysis. Except for water extracts, flower extracts with lower IC_50_ values have a better antiproliferative effect on all cells tested than leaf extracts. Methanol extracts possessed apoptotic and cell cycle arrest potential in HT-29 and Caco-2 human colorectal cancer cells. According to the results of the DPPH, FRAP, and CUPRAC assays, the leaf methanol extract with the highest antioxidant activity was the extract with the highest total phenolic content (29.55 ± 0.66 mgGAE/g extract**)**. The highest total flavonoid (9.19 ± 0.036 mgQE/g extract**)** and total tannin contents (26.45 ± 0.34 mgCE/g extract) were detected in leaf acetone and flower acetone extracts, respectively. The main phenolic compounds identified for leaf methanol (177.192 µg/g) and flower methanol (3885.940 µg/g) extract were naringin and ferulic acid, respectively. The leaf and flower parts possess mineral elements. *V. propontideum* may be considered for pharmacological applications as a promising natural source for new anticancer and antioxidant agents.

**Supplementary Information:**

The online version contains supplementary material available at 10.1007/s11130-026-01470-8.

## Introduction

The disease known as cancer, which is defined by unchecked cell development, is still a major issue worldwide, being among the leading causes of death. Cancer progression is caused by unregulated cell growth and differentiation, as well as the loss of apoptotic activities, which culminate in a large expansion of the neoplastic cell population [1]. Treating cancer has been a very complex process. Cancer treatment methods can be classified into two categories: traditional and modern. While modern approaches include stem cell therapies, anti-angiogenic therapy, immunotherapy, dendritic cell-based immunotherapy, hormone therapy, traditional approaches include radiotherapy, chemotherapy, and surgery. These treatment methods are capable of damage both tumor tissue and normal tissue [2]. In addition, survival rates for people with cancer, especially in advanced stages, remain extremely poor due to high toxicity, medication resistance, and other long-term adverse effects of these treatments [3].

Extremely reactive atoms or groups of atoms that have unpaired electrons in them are known the free radicals. While these radicals take part in biochemical reactions at the cellular level, they can cause cell damage and increased oxidative stress when produced excessively. Oxidative stress is an underlying factor in many health problems. Therefore, it is important to keep free radicals under control and reduce oxidative stress [4]. Antioxidants are ingredients that work to prevent or limit the negative effects of oxidation. These antioxidants are able to neutralize free radicals and protect cells from the harmful effects of oxidative stress. Therefore, antioxidants form an important part of the body’s natural defense mechanisms and play an important role in maintaining cellular health [5].

The enormous chemical diversity in nature indicates that natural products are a rich source of bioactive compounds with potential therapeutic effects [6]. Natural product research is also important as a guide in the development of new drugs. Crude extracts from natural sources can typically enable the isolation of novel and structurally diverse chemical compounds with distinct biological properties [7]. Plants constitute a rich source of bioactive agents due to the secondary metabolites they have. Due to side-effects worries arising from synthetic drugs, plants are being evaluated for their different biological properties assosciated with human health [8].

Extraction with various solvents of different polarity will lead to the presence of different compound classes in the extract. Therefore, examining the extracts obtained with different solvents separately may contribute to the use of plants in pharmaceutical applications [9].

*Verbascum*, belonging to Scrophulariaceae family, is known as mullein. *Verbascum* species have been noted to have traditional medicinal uses such as anti-inflammatory, antimicrobial, anti-diarrheal, expectorant and diuretic, and also have traditional treatment potencies such as for respiratory diseases, dysentery infection, rheumatism, headache and burns [10, 11]. To our best knowledge, there is no comprehensive research in the literature revealing the various biological properties of different extracts of *Verbascum propontideum* Murb.

The current study mainly focused on the determination of different biological properties associated with human health of *Verbascum propontideum* Murb. For the purposes, the extracts were obtained from the leaf and flower parts of the *Verbascum propontideum* Murb. using different solvents. The antiproliferative effects of all extracts on human colorectal adenocarcinoma cell lines (HT-29 and CaCo-2 cells) and human colon noncancerous cell line (CCD18-Co) were evaluated based on concentration and time. The apoptotic effects of methanol extracts on colon carcinoma cells as well as their effects on the cell cycle were investigated. Phenolic compositions of methanol extracts were also investigated by HPLC analysis. Antioxidant potentials and some secondary metabolite amounts (total phenolic, total flavonoid and total tannin) of all extracts were assigned. Additionally, mineral element analyses of leaf and flower parts were performed.

## Materials and Methods

The material and methods section and also some tables and figures has been presented as Supplementary Material.

## Results and Discussion

### Effects of Extracts on Cell Viability

Natural products are the main sources for new therapeutic compounds and have minimal adverse effects. These products have attracted the attention of the pharmaceutical industry, with interest in herbal medicines and alternative treatments [8]. Genetic, environmental and dietary factors play a role in colorectal cancer, which is one of the most frequently diagnosed and fatal malignancies. Plant-based compounds have been known to contribute to the remission of colon cancer in different ways, such as delaying tumor progress and handling the side effects of chemotherapy and radiation therapy. Medicinal plants carry a lot of phytochemicals, such as saponins, flavonoids, triterpenoids, polyphenol compounds, phenols, alkaloids, and others. These compounds with bioactive properties can decrease tumor cell proliferation through various mechanisms, such as arresting the cell cycle and inducing apoptosis [12].

**Fig. 1:**
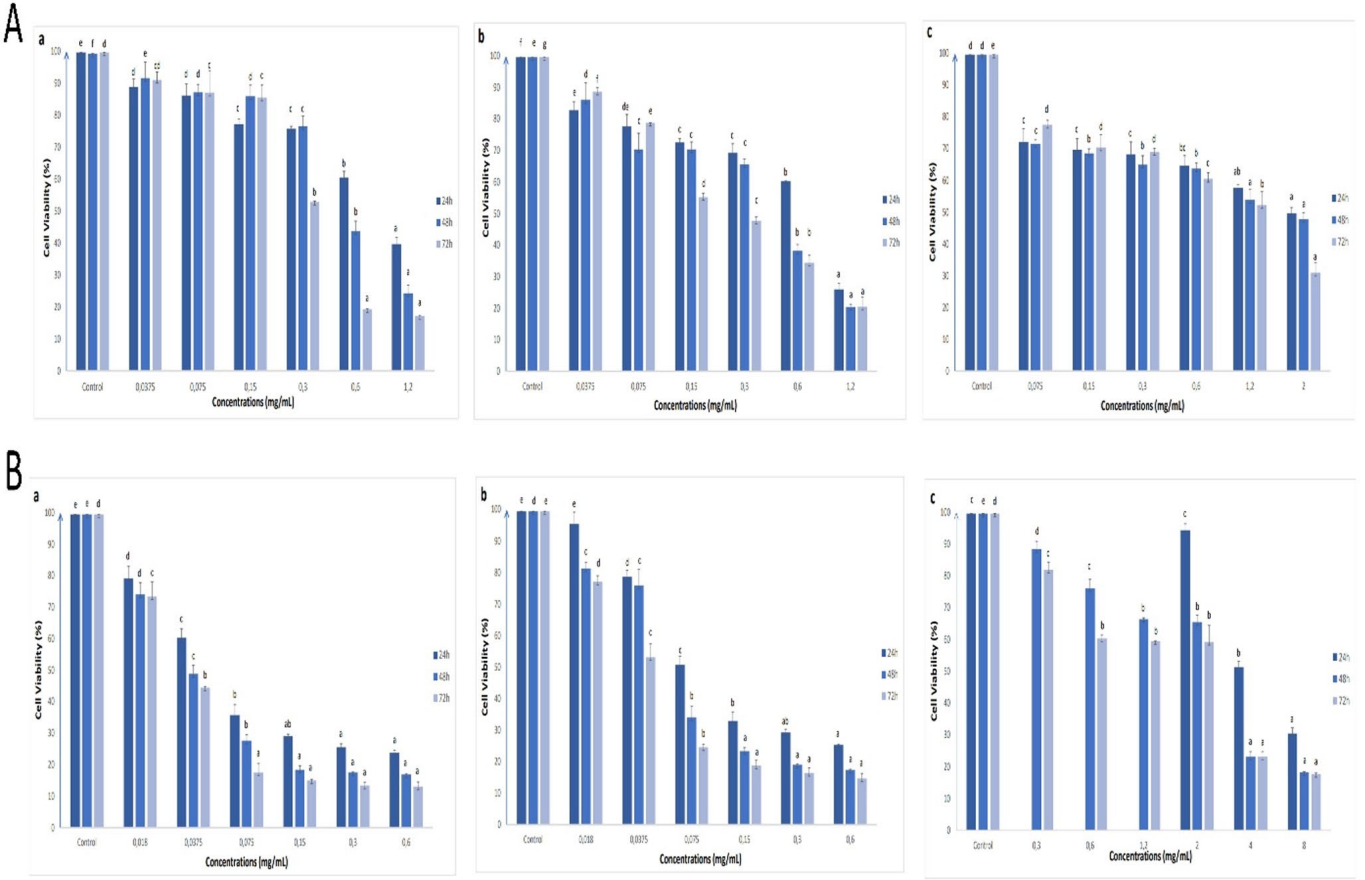
The effects of methanol (**a**), acetone (**b**) and water (**c**) extracts of leaf (**A**) and flower (**B**) parts of *V. propontideum* on the cell viability of HT-29 cells depending on concentration and time. The results were given as mean ± standard error. For statistical analysis of the results of the MTT assay for each incubation time, one-way ANOVA and followed by Duncan’s multiple range test were performed. Different letters indicate significant differences (*p* < 0.05)

**Fig. 2:**
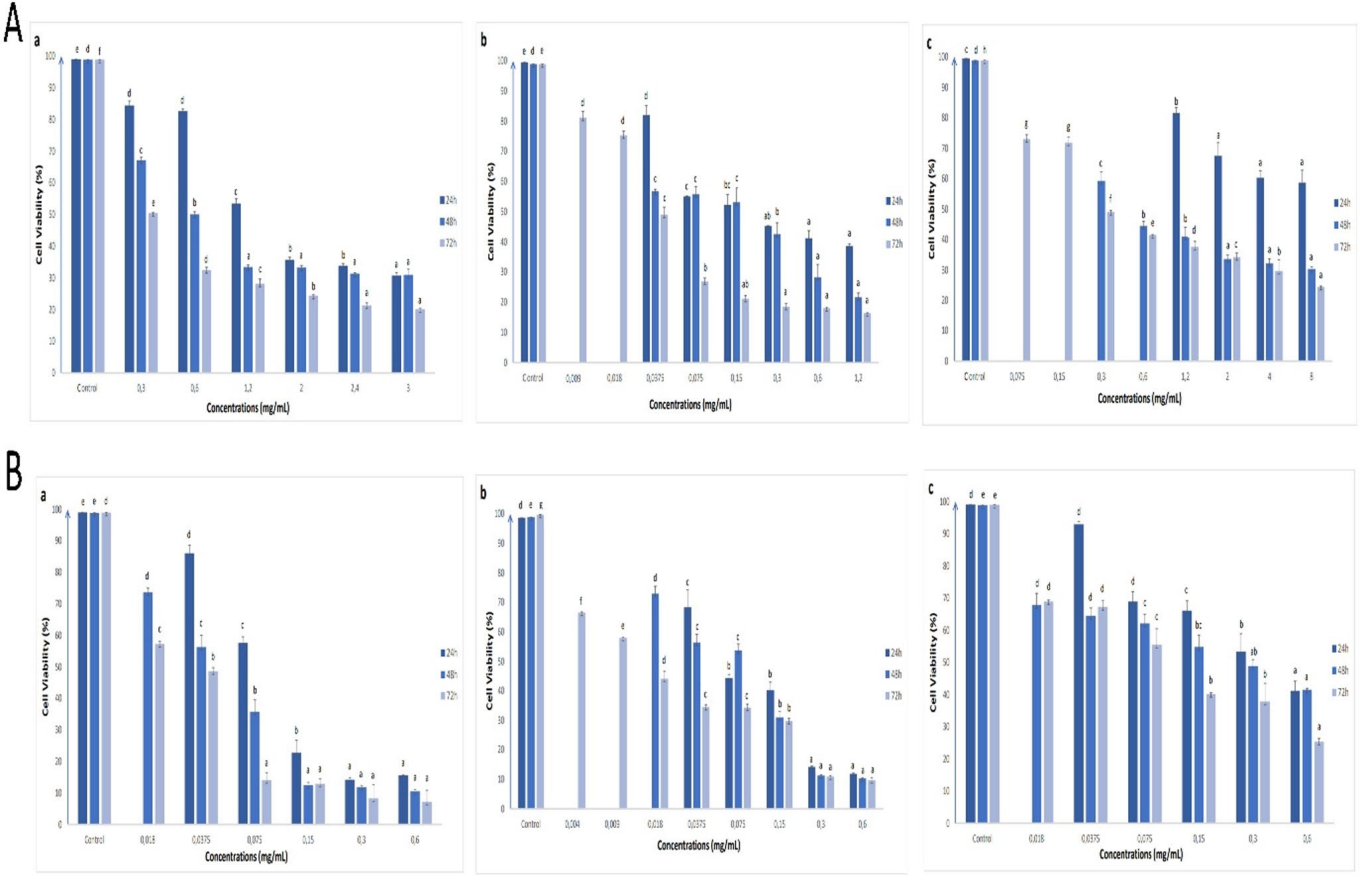
The effects of methanol (**a**), acetone (**b**) and water (**c**) extracts of leaf (**A**) and flower (**B**) parts of *V. propontideum* on the cell viability of CaCo-2 cells depending on concentration and time. The results were given as mean ± standard error. For statistical analysis of the results of the MTT assay for each incubation time, one-way ANOVA and followed by Duncan’s multiple range test were performed. Different letters indicate significant differences (*p* < 0.05)

**Fig. 3:**
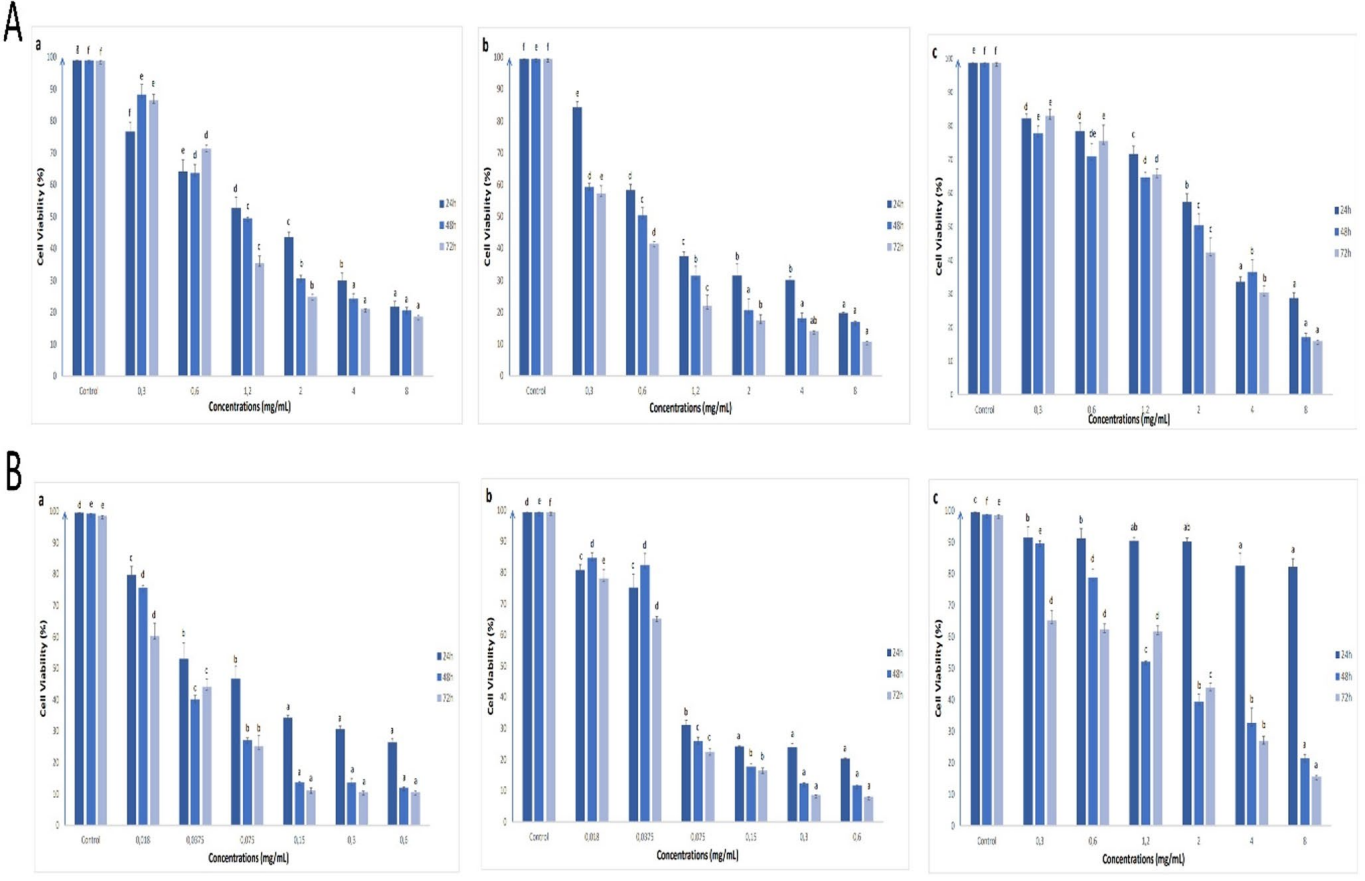
The effects of methanol (**a**), acetone (**b**) and water (**c**) extracts of leaf (**A**) and flower (**B**) parts of *V. propontideum* on the cell viability of CCD18-Co cells depending on concentration and time. The results were given as mean ± standard error. For statistical analysis of the results of the MTT assay for each incubation time, one-way ANOVA and followed by Duncan’s multiple range test were performed. Different letters indicate significant differences (*p* < 0.05)

The potential antiproliferative and apoptotic effects of different extracts of *V. propontideum* were assessed for the first time. These extracts generally exhibited the antiproliferative effects on human colon cell lines tested, depending on time and concentration (Figs. 1, 2 and 3). Considering the approximate IC_50_ values (Table S1), it can be said that flower extracts with lower IC_50_ values have a better antiproliferative effect on all cells tested than leaf extracts, except for water extracts.

In a previous study, the cytotoxic activities of extracts obtained from the leaves and roots of *V. insulare* with ethanol and purified water were examined against HT-29, MCF-7 (human breast cancer), and L-929 (mouse fibroblast) cell lines, and ethanol extracts have been found to show higher antiproliferative activity than purified water extracts [13]. When methanol extracts obtained from *V. cheiranthifolium* var. *asperulum*, *V. pynostachyum* and *V. orgyale* were compared in terms of cytotoxic effects, it was shown that *V. pynostachyum* extract had a promising toxic effect on both HeLa (human cervical) and Skov-3 (human ovarian) cancer cell lines compared to the other species [14]. The extracts prepared with diverse solvents appear to have different antiproliferative effects depending on the species or parts of the *Verbascum* genus tested and the cell line used.

### Apoptotic Effects of Extracts

In cancer treatment, targeting apoptotic pathways is an important approach for eliminating cancer cells through apoptosis [15]. According to the results of the current investigation (Fig. S1 and S2), the percentages of total apoptotic cells (quadrants 2 and 4) in both cell lines were determined to increase with increasing concentrations of the methanol extracts of leaf and flower parts of *V. propontideum* for 24 h compared to control, with the increases in cells treated with approximately 2xIC_50_ and IC_50_ concentrations being particularly noticeable, suggesting that these extracts have the potential to induce apoptosis. Vasincu et al. [16] reported that two compounds, which are “verbascoside and 6-O-(3″,4″-di-O-trans-cinnamoyl)-α-l-rhamnopyranosylcatalpol (Dicinn)”, obtained from *V. ovalifolium* had the potential to induce apoptosis.

### Effects of Extracts on Cell Cycle

While the activity of cell-cycle proteins is tightly controlled in normal cells, it is often dysregulated in human cancer cells. It has been reported that targeting cell cycle machinery may provide an essential way to enhance the effectiveness of cancer treatment [17]. The results of the present research (Fig. S3 and S4) suggest that the *V. propontideum* methanol extracts, whose apoptotic effects were investigated, have the potential to arrest the cell cycle. Treatment of both cell lines with different concentrations of leaf methanol extracts resulted in a notable increase in the percentage of cells in the G2 phase. In a study, Dicinn obtained from *V. ovalifolium* was reported to induce cell-cycle arrest at G0/G1 phase [16].

### Antioxidant Activities of Extracts

Free radicals can interact with other biological molecules in the environment and disrupt their biological structure. Antioxidants are critical molecules that have the capability of preventing the formation of free radicals, whilst also scavenging and neutralizing or repairing the damage they cause [5]. Different parts of plants can contain different secondary metabolites as sources of important natural bioactive compounds. The synthesis of secondary metabolites in plants may vary depending on many factors such as geographical conditions, climatic conditions, and the amount of sunlight [18].

The antioxidant potential of the extracts, which was shown for the first time in this study, varied slightly in different tests performed to evaluate their antioxidant activities. In that, when a general evaluation was made among the extracts, the best antioxidant activity was observed in the leaf methanol extract in three experiments (DPPH, FRAP, CUPRAC), while it was seen in the leaf acetone extract in the another experiment (ABTS). According to the *β*-carotene-linoleic acid method, of all the extracts, the leaf acetone and the flower methanol extracts showed as much antioxidant activity as BHA at 1 mg/mL (Table S2). Also, Angeloni et al. [19] reported that the antioxidant capacity of the extracts of *V. bombyciferum* was relatively varied in different antioxidant assays. According to the IC_50_ values in Table S2, the DPPH free radical scavenging activity of the leaf methanol extract of *V. propontideum* was higher than that of Yabalak et al. [20], who stated that IC_50_ values for DPPH radical scavenging activity of methanol, ethanol and water extracts of above-ground parts of *V. pseudoholotrichum* were calculated as 0.775, 1.254 and 0.539 mg/mL, respectively, and also higher than that of Aydin et al. [21], who reported that IC_50_ values of DPPH analysis were 1.220 ± 0.025 and 2.698 ± 0.013 mg/mL for ethanol and acetone extracts of *V. glomeratum*, respectively. When leaf and flower parts were evaluated separately in the present study, the DPPH radical scavenging activity of methanol extracts was found to be higher than that of acetone extracts. Hazman et al. [22] expressed that the methanol extract of *V. lasianthum* was better than its acetone extract in terms of DPPH radical scavenging activity. The ABTS free radical scavenging activity of leaf acetone extract in the present study was better than ethanol (IC_50_ 2.223 ± 0.014 mg/mL) and acetone (IC_50_, 1.552 ± 0.021 mg/mL) extracts of *V. glomeratum* reported by Aydin et al. [21]. Similar to our study, Angeloni et al. [19] stated that the methanolic extracts of *V. bombyciferum* showed the strongest reducing power in both FRAP and CUPRAC assays; however, their extracts were more potent than the extracts in the current study in terms of these powers. The reducing powers reported for the different extracts of *V. oocarpum* and *V. euphraticum* were higher than those in the present study [23]. In addition, in the present study, the findings of *β*-Carotene/linoleic acid assay for the extracts were higher than those of Aydin et al. [21].

The importance of solvent polarity on phenolic contents and biological activities of extracts has been noted [9]. So, the findings presented herein support that the observed differences in antioxidant activities in plant extracts may be due to the polarity of the solvents used, and the variations among plant species.

### Quantitative Analysis of Extracts

The antioxidant activity observed in the extracts might be attributed to their phenolic contents [19, 22, 23]. In the present study, the leaf methanol extract was the extract with the highest total phenolic content, and the highest total flavonoid content was determined in the leaf acetone extract. In terms of total tannin content, the flower acetone extract was the richest (Table S3). While the extracts in this study had less total phenolic content than those of methanolic extracts of *V. nudicaule* (72.95 ± 0.33 mg GAE/g dry weight), *V. speciosum* (95.83 ± 1.39 mg GAE/g dry weight), and *V. sinuatum* (118.2 ± 2.46 mg GAE/g dry weight) from Iran, the acetone extracts in the present study were richer than these extracts (4.83 ± 0.13, 4.87 ± 0.06, and 5.77 ± 0.23 mg QE/g dry weight, respectively) in terms of total flavonoids [24]. Alkowni et al. [25] showed in their study that among *V. fruticulosum* extracts, the methanol extract had the highest tannin content (120.74 ± 7.14 mg of CAE/g), while the acetone extract had the highest total phenol (64.70 ± 1.60 mg of GAE/g) and flavonoid content (59.84 ± 0.36 mg of RUE/ g).

The results of Pearson’s correlation analysis performed to evaluate the relationship between total secondary metabolite amounts (total phenolic, total flavonoid and total tannin contents) and antioxidant activity assays are shown in Fig. S5. A strong positive correlation was found between total phenolic content and antioxidant activities determined by FRAP and CUPRAC assays. The negative correlations observed between total phenolic content and the IC_50_ values of DPPH and ABTS assays can be interpreted as indicating that total phenolic content is inversely proportional to the IC_50_ value of antioxidant activity, as noted by Abduh et al. [26]. Likewise, the negative correlation observed between total flavonoid content and ABTS can be interpreted in a similar way. As a matter of fact, in this study, it was determined that the leaf methanol extract with the highest antioxidant activity according to DPPH, FRAP, CUPRAC assays had the highest total phenolic content, and the leaf acetone extract with the highest ABTS activity had the highest total flavonoid content. A high degree of correlation between total phenolic content and FRAP and CUPRAC analyses was also emphasized in a study on *V. bombyciferum* extracts [19].

### HPLC Analysis of Extracts

Phenolic compounds occur naturally in plants, help combat oxidative stress, and also have beneficial health effects. The detection of phenolic compounds in plants remains current in terms of their evaluation as new phenolic compound sources [27]. HPLC analysis was performed to determine the phenolic composition of the methanol extracts of *V. propontideum*. The corresponding chromatograms are presented in Fig. S7. According to the results of HPLC analysis (Table 1), fifteen phenolic compounds were identified in both extracts, the primary phenolic compounds identified in the flower methanol extract were ferulic acid (3885.94 µg/g extract) and naringin (897.027 µg/g extract). Furthermore, naringin (177.192 µg/g extract), followed by rutine (120.715 µg/g extract) and ellagic acid (104.879 µg/g extract) were detected to be the most abundant compound in the leaf methanol extract.

**Table 1:** Phenolic composition of methanol extracts of *V. propontideum* (µg/g extract)

No	Phenolic Compounds	RT (min)	UV_max_ (nm)	LOD (µg/mL)	Leaf Methanol (µg/g )	Flower Methanol (µg/g )
1	Gallic acid	6.8	280	0.01	0.002	0.032
2	3,4-dihydroxybenzoic acid	10.7	280	0.03	0.006	0.001
3	4-hydroxybenzoic acid	15.7	280	0.01	0.321	1.282
4	2,5 dihydroxybenzoic acid	17.2	320	0.75	10.832	14.370
5	Chlorogenic acid	18.2	320	0.01	0.629	21.661
6	Vanilic acid	19.2	320	0.11	4.897	0.960
7	Epicatechin	21.3	260	0.43	0.398	48.979
8	Caffeic acid	22.7	280	0.01	18.300	31.975
9	*p*-Coumaric acid	26.1	320	0.01	0.574	0.062
10	Ferulic acid	30.1	320	0.01	11.877	3885.940
11	Rutin	45.6	360	0.57	120.715	74.171
12	Ellagic acid	47.7	240	0.45	104.879	30.446
13	Naringin	49.7	280	0.40	177.192	897.027
14	Cinnamic acid	67.8	280	0.01	12.212	5.912
15	Quercetin	71.1	360	0.57	6.305	1.378

*RT* retention time, *LOD* limit of detection

Ferulic acid (FA) is a non-toxic compound known for its wide range of biological effects, including antibacterial, anti-inflammatory, antidiabetic, immunostimulant, and anticancer properties. It has also been reported to may help heal damaged nerve cells, and to have reduce inflammation. The pharmaceutical industry employs FA as both an antioxidant and an anti-inflammatory agent. In addition to this, it is utilised in the food industry for a number of purposes, including the edible packaging films and food preservatives. It also serves as a photoprotective agent and brightening ingredient in skin products [28]. Naringin, known to have several functions such as anti-cancer, antioxidant, cholesterol-lowering, anti-atherosclerosis, and metal-binding properties, has been declared to enhance the metabolism and absorption of drugs [29]. In the present study, considering the IC_50_ values, the flower methanol extract’s greater antiproliferative activity compared to the leaf methanol extract may have been due to its richer content of these two compounds. In a previous study conducted to determine the phenolic acid profiles of extracts and fractions of *V. anisophyllum* and *V. davidoffi*, the main phenolic acid in all extracts and fractions was reported to be ferulic acid [30]. Unlike current study, ferulic acid was identified as 75.23 µg/g dry weight in *V. pestalozzae* and 4.96 µg/g dry weight in *V. myriocarpum*, but was not detected in *V. detersil* and *V. bellum* [31].

### Mineral Analysis of *Verbascum propontideum*

The presence of a variety of phytochemical and elemental contents donates to the therapeutic impressions of plants, which are rich sources of components needed by humans. Therefore, studies to determine the contents of plants are important [32]. Minerals play an important role in the maintenance of certain physicochemical processes necessary for life. Minerals are usually classified as macro- and micro-minerals. Macrominerals contain the elements such as “potassium (K), phosphorus (P), calcium (Ca), sodium (Na), magnesium (Mg), and sulfur (S)”, while microminerals contain elements such as “copper (Cu), iron (Fe), manganese (Mn), boron (B), zinc (Zn), molybdenum (Mo), selenium (Se), and chromium (Cr)” [33].

Calcium (Ca) is essential for various vital processes, including skeletal stability, blood clotting, neuromuscular functions, and the transmission of hormonal signals to target organs via intracellular signaling pathways. Magnesium (Mg) acts as a co-factor of many enzymes involved in processes such as RNA and DNA synthesis, protein synthesis, energy metabolism, and maintenance of the electrical potential of cell membranes [33, 34]. Potassium (K), essential for the proper functioning of tissues and organs, is necessary for heart function, normal digestion, and muscle function. Phosphate, a key component of bone mineral, and plays a role in meeting skeletal needs. Phosphorus (P) is also a prevalent constituent of DNA, RNA, and cell membrane structure, manifesting in the form of phospholipids, and involved in ATP synthesis, the primary source of energy, and also important for the process of phosphorylation of various proteins and sugars [33]. The general order of abundance of macroelements in *V. propontideum* was Ca > K> Mg and K > Mg> Ca for leaf and flower parts, respectively, in addition, P content was determined to be the same for both parts (Table 2). Iron (Fe), one of the essential elements that supports a healthy immune system and participates in the structure of hemoglobin, responsible for oxygen transport, and catalase, an important antioxidant enzyme for the immune system. Copper (Cu), which is important for iron absorption, contributes to various physiological processes, including the antioxidant protection, regulation of immune functions, cholesterol and glucose metabolism, and formation of myelin. It has been reported that manganese (Mn), an element that can ensure normal growth and development and maintain cellular homeostasis, can be associated with therapeutic properties against cardiovascular and diabetic diseases. Zinc (Zn), which supports the antioxidant system, plays a role in many biological processes, such as growth, protein synthesis, immune system function, cellular respiration, and redox processes [22, 34]. The general order of abundance of microelements in *V. propontideum* was Fe > Zn> Mn > B> Cu for both leaf and flower parts (Table 2). Fe was the most abundant microelement in both plant parts in the current investigation, and was found at a higher concentration in leaf part than in flower part.

**Table 2:** Mineral analysis of *V. propontideum*

Mineral content	Leaf part	Flower part
Phosphorus (P) (%)	0.01	0.01
Potassium (K) (%)	0.6	0.94
Calcium (Ca) (%)	0.83	0.39
Magnesium (Mg) (%)	0.42	0.46
Iron (Fe) (ppm)	383.54	262.71
Copper (Cu) (ppm)	2.51	5.79
Manganese (Mn) (ppm)	26.83	21.06
Zinc (Zn) (ppm)	48.82	44.53
Boron (B) (ppm)	22.22	17.68

The Fe and Mn contents of the aerial parts of *V. orientale* collected at the flowering stage were reported as 225.05 and 22.74 mg/kg, respectively; furthermore, unlike the present study, the Cu and Zn contents were noticed as 18.26 and 29.81 mg/kg, respectively [35]. Zn and B contents found in the present investigation were observed to be comparatively higher than those in the *V. lasianthum* (a mean of 20.67 and 0.14 ppm, respectively) presented by Hazman et al. [22]. Being aware of the concentrations of elements in plants is crucial for comprehending their pharmacological effects and evaluating their therapeutic potential [34].

## Conclusion

It is our considered opinion that, this study is the first detailed investigation on the evaluation of different biological activities of the extracts of leaf and flower parts of *V. propontideum*. Depending on concentration and time, extracts generally reduced cell viability. The methanol extracts of leaf and flower parts were found to have the ability to increase the percentage of apoptotic cells and to cause cell cycle arrest in HT-29 and Caco-2 cells when compared to the control. Although antioxidant activity values varied depending on the experiments used, it may be said that the leaf methanol extract, which had the highest phenolic content among all the extracts tested, generally had good antioxidant activity. The highest total flavonoid and tannin contents were determined in leaf acetone and flower acetone extracts, respectively. Ferulic acid was the main phenolic compound, which was detected in the flower methanol extract. Fe was the most abundant microelement in leaf and flower parts. Considering the data obtained, *V. propontideum* may be considered for pharmacological applications as a natural source for new anticancer and antioxidant agents. It is expected that the findings will provide basic data for further studies to obtain more detailed information about *V. propontideum*. It would be useful to evaluate research on the purification and identification of the compounds with bioactive properties from the plant. However, further research including comprehensive toxicological assessment, bioavailability studies, and clinical in vivo studies is recommended to evaluate the plant’s diverse potential functional uses such as in pharmaceuticals, nutraceuticals, and cosmetics as well as its biological properties reported here.

## Supplementary Information

Below is the link to the electronic supplementary material.

Supplementary Material 1 (DOCX 793 KB)

## Data Availability

No datasets were generated or analysed during the current study.
