# Supplementary material for: A First Investigation on Verbascum propontideum Murb.: Comparative Biological Properties, Phytochemical Profile and Mineral Composition
Source: Plant Foods Hum Nutr. 2026 Feb 17;81(1):21. doi: 10.1007/s11130-026-01470-8 (PMC12909325; doi:10.1007/s11130-026-01470-8)
Supplement: Supplementary file 1 — Supplementary Material 1 (DOCX 793 KB) [file 11130_2026_1470_MOESM1_ESM.docx]

**Plant Foods for Human Nutrition**

**A first investigation on *Verbascum propontideum* Murb.: comparative biological properties, phytochemical profile and mineral composition**

**Burcu Şahin^1^**

[brcshn8@icloud.com](mailto:brcshn8@icloud.com)

https://orcid.org/0000-0003-0005-8539

**Mehlika Alper^1^***

[mehlikaalper@mu.edu.tr](about:blank)

[https://orcid.org/0000-0001-6193-346X](about:blank)

^1^ Department of Molecular Biology and Genetics, Faculty of Science, Muğla Sıtkı Koçman University, 48000, Kötekli, Muğla, Turkey

*: **Corresponding Author**: Mehlika Alper

**Supplementary Material**

Materials and Methods

Reagents and chemicals

Reagents, chemicals and mediums for cell culture were provided from Capricorn Scientific (GmbH, Germany), Applichem (Germany), and Biochrom (GmbH, Germany). Other reagents and chemicals for antioxidant assays were obtained from Sigma-Aldrich (Germany) or Merck (Germany).

Collection and identification of plant

*V. propontideum* Murb. was collected in June 2021 from the rocky slopes of the roadside on the Marmaris-Datça D400 highway in Muğla province (Turkey), 80 m, 36^0^7'50''N, 28^0^4'27''E coordinates and identified by expert taxonomist Olcay Ceylan (PhD) from Muğla Sıtkı Koçman University (herbarium number of the voucher specimen in their herbarium: O.2135).

Extract preparation

The leaves and flowers of *V. propontideum* were seperately dried in a shade and airy environment. The extraction process was performed according to the method given by Turan and Mammadov [1], using acetone, methanol, and distilled water as solvents. The extracts obtained after the lyophilization process were stored at -20 °C, protected from light.

Cell culture and MTT assay

“Roswell Park Memorial Institute (RPMI)-1640 medium”, enriched by adding 10% Fetal bovine serum (FBS) and penicillin-streptomycin (100 U/ml-100 μg/ml) antibiotic, was used as the growth medium for the human colorectal adenocarcinoma cell lines (HT-29 and CaCo-2 cells, American Type Culture Collection) and human colon noncancerous cell line (CCD18-Co, American Type Culture Collection). Cells were grown in an incubator providing 5% CO_2_ and 95% humidity at 37 °C.

The effects of the extracts on concentration and time-dependent cell viability were determined based on the “MTT (3-(4,5-dimethylthiazole-2)-2,5-diphenyltetrazolium bromide)” assay [2], with some modifications, also as mentioned in earlier research by Güneş et al. [3], and Ardıl and Alper [4]. MTT test was performed at 2x10^4^ cells/well for all cell lines and for three incubation times (24, 48 and 72 hours). Cell viability (%) was calculated taking into account the formula stated by Cabus et al. [5].

Apoptosis detection by flow cytometry

The apoptotic effect of methanol extracts on HT-29 and CaCo-2 cell lines was determined using flow cytometry according to an “Annexin V-FITC Apoptosis Detection Kit” (Elabscience E-CK-A211) protocol. The apoptotic effect of the extracts on cells was investigated at concentrations of approximately 2xIC_50_, IC_50_ and ½xIC_50_ for 24 hours. Cells not treated with the extract served as control. Analysis was performed using the Flow cytometry device (BD FACSCanto A, BD Biosciences, USA) and the BD FACSDiva (v6.13) program.

Analysis of cell cycle by flow cytometry

The effects of methanol extracts, whose apoptotic effect was investigated, on the cell cycle in HT-29 and CaCo-2 cell lines were analyzed using flow cytometry for concentrations of approximately 2xIC_50_, IC_50_ and ½xIC_50_ of the extracts for 24 hours. The analysis was completed via flow cytometry using ModFit LT 3.0 software [6]. The experimental protocol followed was also given by Ardıl and Alper [4].

Antioxidant Activity Assays

*DPPH and ABTS radical scavenging assays*

DDPH (2,2-Diphenyl-1-picrylhydrazyl) free radical scavenging and ABTS (2,2-Azino-bis3-ethylbenzothiazoline-6-sulfonic acid) radical cation scavenging activities were investigated based on the method reported by Turan and Mammadov [1], and the IC_50_ (50% inhibition concentration) values were calculated. BHA (Butylated Hydroxyanisole) was positive control for these two experiments.

***FRAP and CUPRAC assays***

Based on the method of Benzie and Strain [7] with slight modifications, ferric reducing antioxidant power (FRAP) assay was performed. 2 mL of FRAP reagent solution (“10 mM TPTZ (2,4,6-tripyridyl-s-triazine), 0.3 M acetate buffer (3.6 pH”), and 20 mM FeCl_3_( ferric chloride)) was added to the extract solution prepared in 1 mg/mL own solvent. After 30 minutes incubation, the absorbance was read at a wavelength of 595 nm. In the light of the method developed by Apak et al. [8] with slight modifications, cupric reducing antioxidant capacity (CUPRAC) was determined. The extract solution (0.5 mL, 1 mg/mL) was added onto the CUPRAC solution (“1 mL of ammonium acetate, 1 mL of CuCl_2_ (copper(II) chloride), 1 mL of neocuproine”). After 30 minutes incubation, absorbance values were measured at a wavelength of 450 nm. The calibration charts were created after the experiments performed using Trolox (0.05-0.2 mg/mL for FRAP assay; 0.01-0.05 mg/mL for CUPRAC assay). The outcomes were given as trolox equivalent (mg TE/g extract).

***β-Carotene-linoleic acid assay***

The β-Carotene-linoleic acid method was performed by following the procedure described by Amarowicz et al. [9]. In brief, a β-carotene stock solution was prepared by dissolving “2 mg of β-carotene in 1 mL of chloroform”. “Linoleic acid (0.02 mg) and Tween 20 ( 0.2 mg)” were added to 1 mL of this stock solution. After chloroform evaporation, 100 mL of dH_2_O was added and mixed. 24 mL of this mixture was added to test tubes containing 1 mL of extract solution. The initial absorbances were immediately measured at 470 nm using a spectrophotometer. The tubes were incubated at 50 °C. Incubation was maintained until “the color of β-carotene disappeared” (120 minutes). The test tube with BHA was positive control. The antioxidant activity was calculated as a percentage using the formula given by Amarowicz et al. [9].

Quantitative analysis

***Determination of total phenolic content***

In the light of the method of Singleton and Rossi [10], to estimation of total phenolic content, “FCR (Folin-Ciocalteu Reagent)” were used. The extract (1 mg/mL), Folin-Ciocalteu reagent and dH_2_O (1 mL: 1 mL: 46 mL) were mixed. After 3 minutes, 2% sodium carbonate (Na_2_CO_3_) solution (3 mL) was added. After for 2 hours darkly incubation, and measurement was made at 760 nm. A calibration chart using gallic acid (0.01-0.05 mg/mL) was drawn. Using the equation obtained from this graph, the outcomes were expressed as gallic acid equivalent (mg GAE) per g of extract.

***Determination of total flavonoid content***

Total flavonoid content was evaluated using the method determined by Aryal et al. [11]. 0.2 mL of sodium acetate and then 1 mL of aluminum chloride were added to the extract solution (1 mg/mL) and shaken. After adding 5.6 mL of distilled water and incubating for 30 minutes, the optical density was read at 415 nm. A calibration chart was created using quercetin (0.01-0.05 mg/mL). With the help of the equation obtained from this graph, the outcomes were presented as quercetin equivalent (mg QE/g extract).

***Determination of total tannin content***

The total tannin content was detected according to procedure developed by Bekir et al. [12]. Vanillin reagent (“1.5 mL, 1% in 7 M H_2_SO_4_”) was added to the extract solution (0.5 mL, 1 mg/mL) and mixed in a ice bath. After 15 minutes, the optical density at 500 nm was read. A calibration chart was created using catechin (0.01-0.05 mg/mL). At the end, the outcomes were expressed as catechin equivalent (mg CE) per g of extract from the calibration graph drawn according to the absorbance values obtained.

HPLC analysis

Analysis of phenolic composition was performed by “Reversed-phase High Performance Liquid Chromatography” (RP-HPLC, Shimadzu, Japan) according to the protocol described by of Caponio et al. [13] with some modifications. The details of this procedure were given in Alper and Güneş [14]. The standard HPLC chromatogram (Fig. S6) shown in this study was taken from our previously published work [15], as the analyses were performed under identical experimental conditions. The quantity of each compound was given as μg/g extract.

Mineral element analysis

The analysis of mineral elements was performed using ICP-OES (“optical emission spectrometer with inductively coupled plasma”) method. This analysis was carried out by Muğla Sıtkı Koçman University Research Laboratories Center (Muğla Agricultural Soil, Plant and Irrigation Water Analysis Laboratory) within the scope of service procurement.

Analysis of Data

Statistical analyses and IC_50_ calculations were performed using SPSS statistical software (version 22.0) (IBM, USA). To determine the significant differences, the One-Way analysis of variance (ANOVA) and followed by Duncan's multiple range test were applied (P ≤ 0.05). Also, in this study, Pearson’s correlation analysis was performed to evaluate the correlations between the total amount of secondary metabolites and antioxidant activity assays.

References

1. Turan M, Mammadov R (2018) Antioxidant, antimicrobial, cytotoxic, larvicidal and anthelmintic activities and phenolic contents of *Cyclamen alpinum*. Pharmacology & Pharmacy 9(4): 100-116. <https://doi.org/10.4236/pp.2018.94008>
2. Mosmann T (1983) Rapid colorimetric assay for cellular growth and survival: application to proliferation and cytotoxicity assays. J Immunol Methods 65(1-2): 55-63. <https://doi.org/10.1016/0022-1759(83)90303-4>
3. Güneş H, Alper M, Çelikoğlu N (2019) Anticancer effect of the fruit and seed extracts of *Momordica charantia* L. (Cucurbitaceae) on human cancer cell lines. Trop J Pharm Res 18(10): 2057-2065. http://doi.org/10.4314/tjpr.v18i10.9
4. Ardıl B, Alper M (2022) Potential cancer treatment effects of brusatol or eriodictyol combined with 5-fluorouracil (5-FU) in colorectal cancer cell. Naunyn Schmiedebergs Arch of Pharmacol 395(9): 1109-1123. <https://doi.org/10.1007/s00210-022-02270-y>
5. Cabus U, Secme M, Kabukcu C, Cil N, Dodurga Y, Mete G, Fenkci IV (2021) Boric acid as a promising agent in the treatment of ovarian cancer: molecular mechanisms. Gene 796: 145799. https://doi.org/10.1016/j.gene.2021.145799
6. Yılmazer Çakmak Ö (2011) DNA Fragmentation, Cell Cycle Analysis, Apoptotic Cell Analysis (Annexin V). 1st Theoritical Cell Death research Techniques Course. Book Chapter ISBN: 978 975-441-349-6, pp. 223-232.
7. Benzie IF, Strain JJ (1996) The ferric reducing ability of plasma (FRAP) as a measure of “antioxidant power”: The FRAP assay. Anal Biochem 239(1): 70-76. https://doi.org/10.1006/abio.1996.0292
8. Apak R, Güçlü K, Özyürek M, Esin Karademir S (2004) Novel total antioxidant capacity index for dietary polyphenols and vitamins C and E, using their cupric ion reducing capability in the presence of neocuproine: CUPRAC method. J Agric Food Chem 52(26): 7970-7981. https://doi.org/10.1021/jf048741x
9. Amarowicz R, Pegg RB, Rahimi-Moghaddam P, Barl B, Weil JA (2004) Free-radical scavenging capacity and antioxidant activity of selected plant species from the Canadian prairies. Food Chem 84(4): 551-562. <https://doi.org/:10.1016/S0308-8146(03)00278-4>
10. Singleton VL, Rossi JA (1965). Colorimetry of total phenolics with phosphomolybdic-phosphotungstic acid reagents. Am J Enol Vitic 16(3): 144-158. https://doi.org/10.5344/ajev.1965.16.3.144
11. Aryal S, Baniya MK, Danekhu K, Kunwar P, Gurung R, Koirala N (2019) Total phenolic content, flavonoid content and antioxidant potential of wild vegetables from Western Nepal. Plants 8(4): 96. https://doi.org/10.3390/plants8040096
12. Bekir J, Mars M, Souchard JP, Bouajila J (2013) Assessment of antioxidant, anti-inflammatory, anti-cholinesterase and cytotoxic activities of pomegranate (*Punica granatum*) leaves. Food Chem Toxicol 55: 470-475. https://doi.org/10.1016/j.fct.2013.01.036
13. Caponio F, Alloggio V, Gomes T (1999) Phenolic compounds of virgin olive oil: influence of paste preparation techniques. Food Chem 64(2): 203-209. <https://doi.org/10.1016/S0308-8146(98)00146-0>
14. Alper M, Güneş M (2020). Evaluation of cytotoxic, apoptotic effects and phenolic compounds of sea cucumber Holothuria tubulosa (Gmelin, 1791) extracts. Turk J Vet Anim Sci 44(3): 641-655. https://doi.org/10.3906/vet-1909-80
15. Alper M, Özay C, Güneş H, Mammadov R (2021). Assessment of antioxidant and cytotoxic activities and identification of phenolic compounds of *Centaurea solstitialis* and *Urospermum picroides* from Turkey. Braz Arch Biol Technol 64, e21190530. https://doi.org/10.1590/1678-4324-2021190530

**List of Tables**

**Table S1** Approximate IC_50_ values (mg/mL) of *V. propontideum* extracts in the cells

**Table S2** Antioxidant activities of *V. propontideum* extracts

**Table S3** Quantitative analysis of of *V. propontideum* extracts

**Table S1** Approximate IC_50_ values (mg/mL) of *V. propontideum* extracts in the cells.

|  | Cells | | | | | | | | |
| --- | --- | --- | --- | --- | --- | --- | --- | --- | --- |
| Extract | HT-29 | | | CaCo-2 | | | CCD-18Co | | |
|  | 24 h | 48 h | 72 h | 24 h | 48 h | 72 h | 24 h | 48 h | 72 h |
| Leaf Methanol | 0.94 | 0.54 | 0.36 | 1.40 | 0.66 | 0.223 | 1.38 | 1.32 | 1.11 |
| Leaf Acetone | 0.602 | 0.348 | 0.267 | 0.28 | 0.174 | 0.038 | 0.885 | 0.688 | 0.47 |
| Leaf Water | 1.97 | 1.70 | 1.17 | nd^2^ | 0.60 | 0.242 | 2.52 | 1.75 | 1.63 |
| Flower Methanol | 0.064 | 0.038 | 0.031 | 0.098 | 0.044 | 0.023 | 0.076 | 0.032 | 0.025 |
| Flower Acetone | 0.116 | 0.069 | 0.042 | 0.078 | 0.058 | 0.019 | 0.071 | 0.064 | 0.047 |
| Flower Water | nd^1^ | 2.07 | 2.59 | 0.428 | 0.251 | 0.099 | nd^3^ | 1.77 | 1.98 |

nd^1^: not determined, because only three concentrations were tried. nd^2^: not determined, because cell viability was over 50% at all concentrations tested. nd^3^: not determined, because cell viability was over 70% at all concentrations tested.

**Table S2** Antioxidant activities of *V. propontideum* extracts

| Sample | DPPH  (IC_50,_ mg/mL)* | ABTS  (IC_50,_ mg/mL)* | β-carotene/linoleic acid (%)* | FRAP  (mg TE/g extract)* | CUPRAC (mg TE/g extract)* |
| --- | --- | --- | --- | --- | --- |
| Leaf Methanol | 0.057±0.002^b^ | 0.028 ± 0.0003^c^ | 71.54±0.34^c^ | 6.13±0.23^a^ | 29.14±0.48^a^ |
| Leaf Acetone | 0.115±0.004^c^ | 0.019 ± 0.0002^b^ | 94.60±0.16^a^ | 2.50±0.03^cd^ | 13.19±0.13^c^ |
| Leaf Water | 0.299±0.038^d^ | 0.061±0.0008^g^ | 47.43±0.84^e^ | 1.90±0.02^e^ | 11.16±0.74^d^ |
| Flower Methanol | 0.296±0.005^d^ | 0.037±0.0002^d^ | 94.28±0.12^a^ | 3.24±0.26^b^ | 18.36±0.43^b^ |
| Flower Acetone | 0.650±0.005^f^ | 0.044 ± 0.0003^e^ | 90.92±0.57^b^ | 2.86±0.07^bc^ | 10.18±0.10^d^ |
| Flower Water | 0.534±0.009^e^ | 0.048±0.0002^f^ | 50.07±0.33^d^ | 2.23±0.08^de^ | 10.24±0.33^d^ |
| BHA | 0.013±0.001^a^ | 0.01±0.001^a^ | 95.77±0.26^a^ | nt | nt |

*: Data were given as mean ± standard error. nt: not tested. Different letters (a-g) in same column denote statistically different from others (*p*<0.05).

**Table S3** Quantitative analysis of of *V. propontideum* extracts

| Extract | Total phenolic content  (mgGAE/g extract)* | Total flavonoid content  (mgQE/g extract)* | Total tannin content (mgCE/g extract)* |
| --- | --- | --- | --- |
| Leaf Methanol | 29.55±0.66^a^ | 4.04±0.002^c^ | 4.98±0.09^c^ |
| Leaf Acetone | 15.10±0.40^cd^ | 9.19±0.036^a^ | 6.82±0.47^b^ |
| Leaf Water | 16.67±0.23^c^ | 1.66±0.009^e^ | 1.98±0.01^d^ |
| Flower Methanol | 16.43±0.38^c^ | 2.25±0.014^d^ | 5.33±0.05^c^ |
| Flower Acetone | 18.79±0.35^b^ | 8.07±0.112^b^ | 26.45±0.34^a^ |
| Flower Water | 14.44±0.33^d^ | 1.65±0.021^e^ | 1.91±0.03^d^ |

*: Data were given as mean ± standard error. Different letters (a-e) in same column denote statistically different from others (*p*<0.05).

**List of Figures**

**Fig. S1** Analysis of apoptosis by flow cytometry in HT-29 cells treated with methanol extracts of leaf (a) and flower (b) parts of *V. propontideum* for 24 h

**Fig. S2** Analysis of apoptosis by flow cytometry in Caco-2 cells treated with methanol extracts of leaf (a) and flower (b) parts of *V. propontideum* for 24 h

**Fig. S3** Cell cycle analysis by flow cytometry in HT-29 cells treated with methanol extracts of leaf (a) and flower (b) parts of *V. propontideum* for 24 h

**Fig. S4** Cell cycle analysis by flow cytometry in CaCo-2 cells treated with methanol extracts of leaf (a) and flower (b) parts of *V. propideum* for 24 h

**Fig. S5** Pearson correlation between the total amount of secondary metabolites and antioxidant activity assays

**Fig. S6** The HPLC standard chromatogram

**Fig. S7** The HPLC chromatogram of methanol extracts of leaf (a) and flower (b) parts of *V. propontideum*


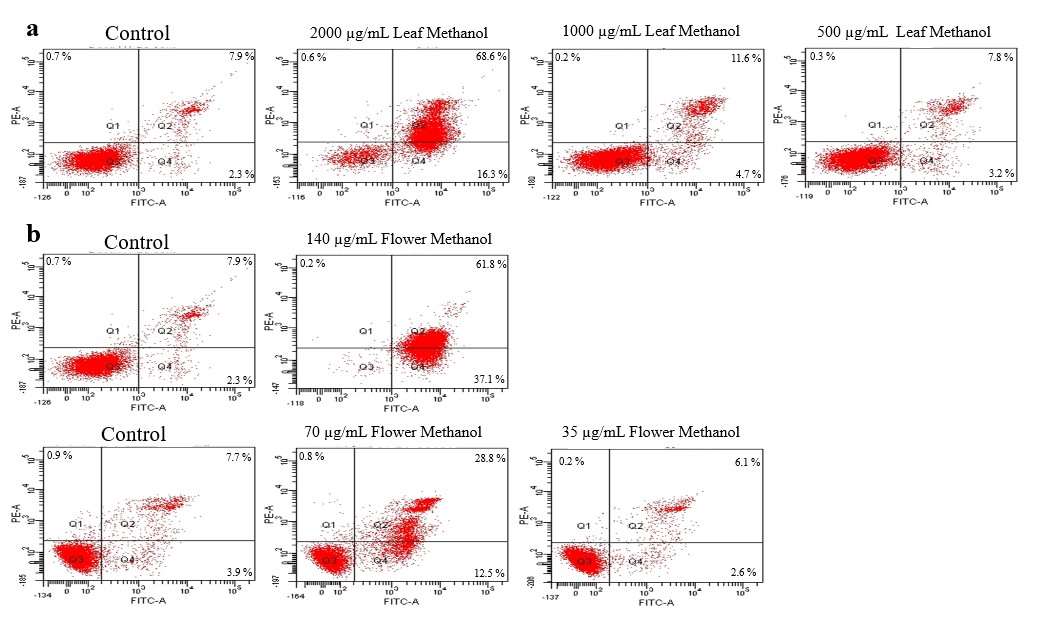


**Fig. S1** Analysis of apoptosis by flow cytometry in HT-29 cells treated with methanol extracts of leaf (a) and flower (b) parts of *V. propontideum* for 24 h. Cells were distributed into four quadrants: Q1-necrotic cells, Q2-late apoptotic cells, Q3-viable cells, and Q4-early apoptotic cells


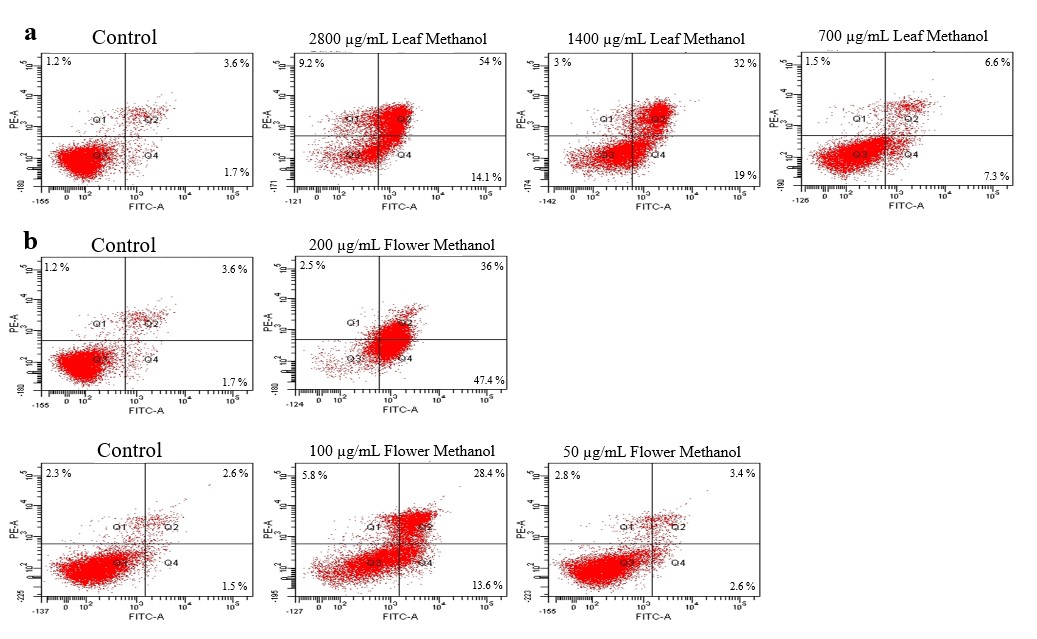


**Fig. S2** Analysis of apoptosis by flow cytometry in Caco-2 cells treated with methanol extracts of leaf (a) and flower (b) parts of *V. propontideum* for 24 h. Cells were distributed into four quadrants: Q1-necrotic cells, Q2-late apoptotic cells, Q3-viable cells, and Q4-early apoptotic cells


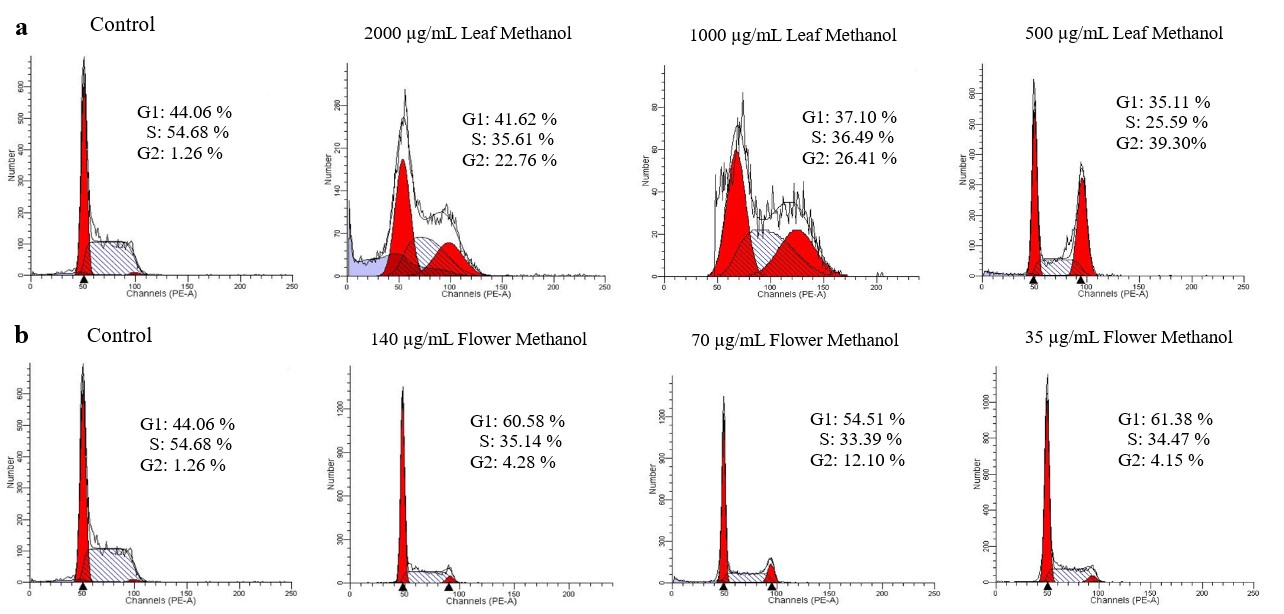


**Fig. S3** Cell cycle analysis by flow cytometry in HT-29 cells treated with methanol extracts of leaf (a) and flower (b) parts of *V. propontideum* for 24 h


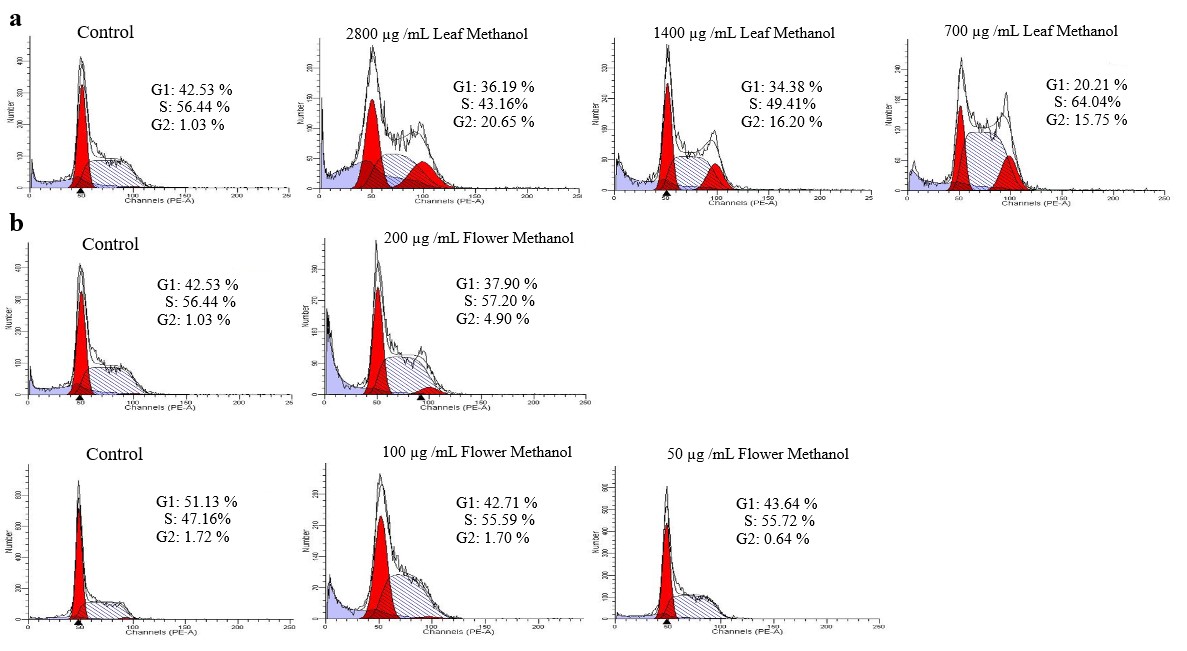


**Fig. S4** Cell cycle analysis by flow cytometry in CaCo-2 cells treated with methanol extracts of leaf (a) and flower (b) parts of *V. propideum* for 24 h


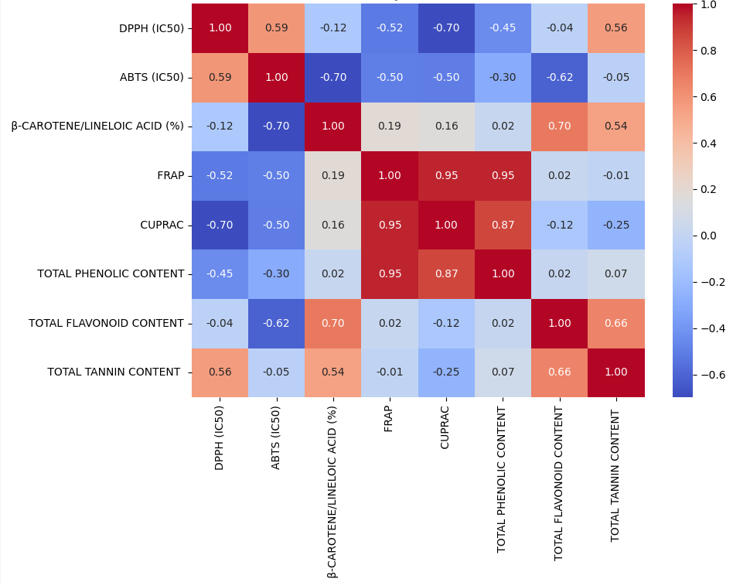


**Fig. S5** Pearson correlation between the total amount of secondary metabolites and antioxidant activity assays. In the color scale representing the correlation coefficient, red color indicates positive correlation and blue color indicates negative correlation


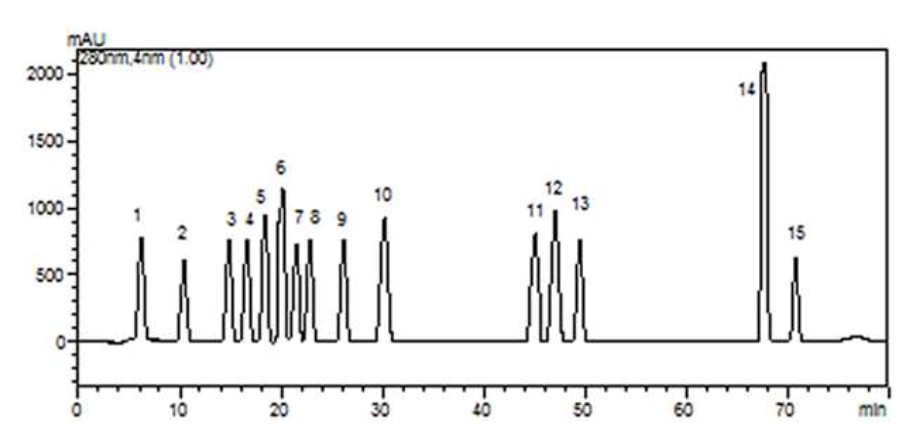


**Fig. S6** The HPLC standard chromatogram


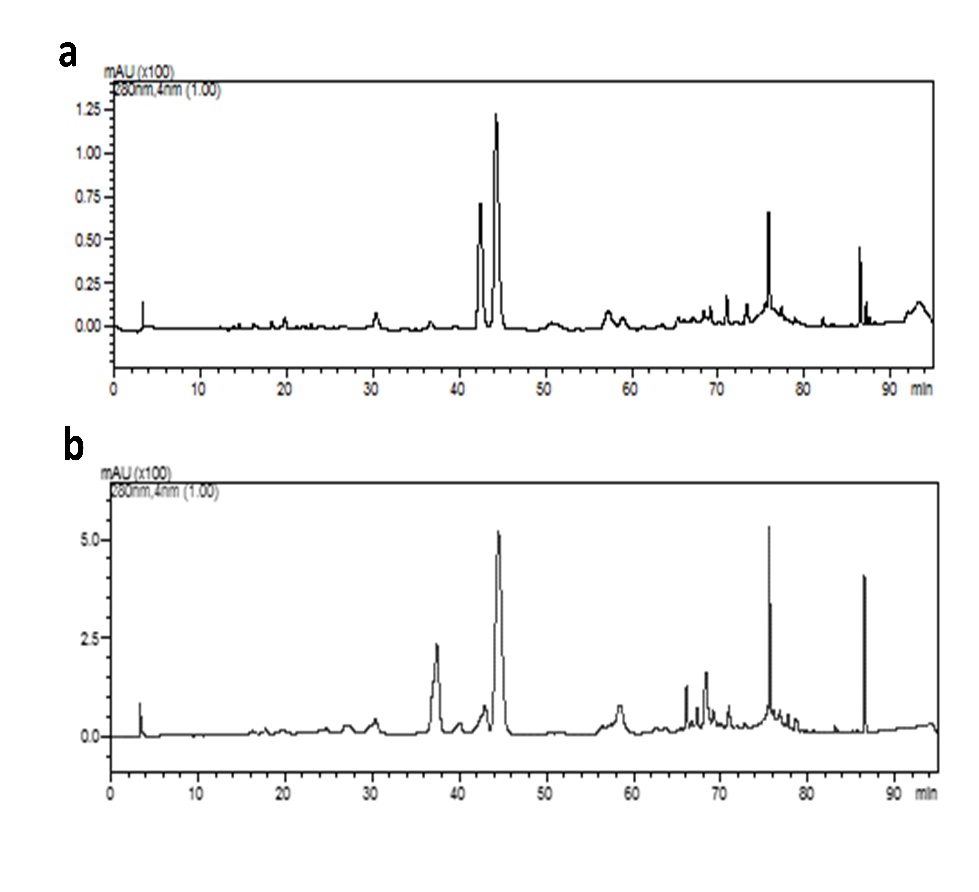


**Fig. S7** The HPLC chromatogram of methanol extracts of leaf (a) and flower (b) parts of *V. propontideum*
